# Hijacking tRNAs From Translation: Regulatory Functions of tRNAs in Mammalian Cell Physiology

**DOI:** 10.3389/fmolb.2020.610617

**Published:** 2020-12-17

**Authors:** Irem Avcilar-Kucukgoze, Anna Kashina

**Affiliations:** Department of Biomedical Sciences, School of Veterinary Medicine, University of Pennsylvania, Philadelphia, PA, United States

**Keywords:** tRNA, tsRNA, arginylation, posttranslational modifications, RNA-mediated signaling

## Abstract

Transfer tRNAs (tRNAs) are small non-coding RNAs that are highly conserved in all kingdoms of life. Originally discovered as the molecules that deliver amino acids to the growing polypeptide chain during protein synthesis, tRNAs have been believed for a long time to play exclusive role in translation. However, recent studies have identified key roles for tRNAs and tRNA-derived small RNAs in multiple other processes, including regulation of transcription and translation, posttranslational modifications, stress response, and disease. These emerging roles suggest that tRNAs may be central players in the complex machinery of biological regulatory pathways. Here we overview these non-canonical roles of tRNA in normal physiology and disease, focusing largely on eukaryotic and mammalian systems.

## Introduction

**FIGURE 1:**
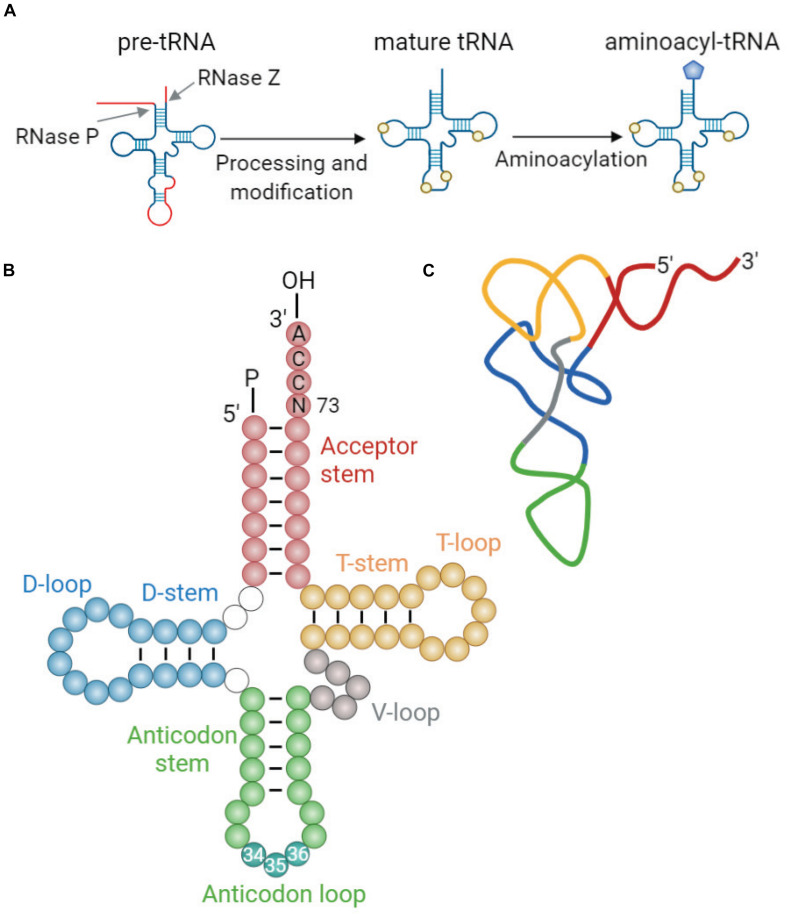
tRNA maturation and structure. **(A)** Maturation of tRNA involves 5′ and 3′ processing, intron splicing, modification of nucleotides, and addition of 3′ CCA end. Mature tRNAs are charged with cognate amino acids by aminoacyl-tRNA synthetases (aaRSs). This reaction involves conjugation of cognate amino acid to the 3′ terminal adenosine of tRNA by an ester bond (Berg and Offengand, 1958). **(B)** The generic secondary structure of tRNA with its constituent domains marked in different colors: acceptor stem (red); dihydrouridine (D-) stem and loop (blue); anticodon stem and loop (green); variable (V-) loop (gray); and TΨC (T-) stem and loop (yellow). Anticodon sequence is depicted in darker blue-green within the anticodon loop and is numbered (nt 34–36). Discriminator base (N73) aiding in aaRS recognition is located upstream of the 3′CCA tail (Giege, 2008). **(C)** Schematic representation of the L-shaped tertiary structure of yeast tRNA^Phe^. Loops are color-coded similarly to those in panel **(B)**. The structure was derived from reported crystal structure (Protein Data Bank code 1EHZ) (Shi and Moore, 2000).

Transfer RNAs (tRNAs) are adaptor molecules that translate genetic information into protein sequence by delivering amino acids to the protein synthesis machinery during translation. Mature tRNAs, formed from pre-tRNA through specialized steps of cleavage and posttranscriptional modifications (Figure 1A), are 73–93 nucleotides (nt) in length. Their secondary structure resembles a cloverleaf shape with four stem-loops: the acceptor stem where the amino acid is attached, the anticodon loop containing the anticodon triplet that recognizes the specific complementary codons on mRNA during protein synthesis, the deoxyuridine stem-loop (D-arm), the TΨC stem-loop (T-arm), and the variable loop (V-loop) (Giege, 2008; Figure 1B). The tRNA cloverleaf further folds into an L-shaped tertiary structure through base pairing of nucleotides in the D-loop and T-loop (Figure 1C).

Transfer RNAs are synthesized as precursors by RNA polymerase III (PolIII) and undergo a multistep maturation process, involving removal of the 5′ leader, trimming of the 3′ trailer, splicing of introns, modifications of nucleotides, and addition of the 3′ terminal CCA tail that serves as a site for amino acid charging (Figure 1A). After processing of pre-tRNAs in the nucleoplasm, mature tRNAs are transported to the cytoplasm through the nuclear pore complexes, which serve as quality control to ensure that only correctly processed tRNAs can pass into the cytoplasm (Chatterjee et al., 2018). In the cytoplasm, mature tRNAs are aminoacylated by their cognate aminoacyl-tRNA synthetases (aaRSs) (Berg and Offengand, 1958), which enables them to deliver the attached amino acids to the ribosome for incorporation into the growing polypeptide chain. This reaction involves conjugation of cognate amino acid to 2′ or 3′ OH of the invariant 3′ terminal adenosine in the tRNA by an ester bond. Each aaRS recognizes specific identity elements within the cognate tRNA, e.g., the anticodon sequence, the discriminator base (N73) located upstream of the 3′ CCA end, and structural elements unique to each particular tRNA (Pang et al., 2014). Following amino acid conjugation, aminoacyl-tRNA complexed with elongation factor (eEF1α in eukaryotes) reaches the ribosome where the peptide bonds are formed.

Each of the 20 amino acids is usually encoded by several codons, which are referred to as synonymous codons. The frequency of synonymous codons is not uniform in the genome. Abundant/preferred codons are represented more than others, while rare/unpreferred codons are less frequent. This phenomenon is known as “codon usage bias.” In prokaryotes and unicellular eukaryotes, the frequency of a codon correlates with the corresponding tRNA gene copy number and the abundance of the cognate tRNA (Ikemura, 1985; Dong et al., 1996; Percudani et al., 1997). This co-adaptation is especially prominent in faster-growing bacteria (Wei et al., 2019), even though the relationship between codon usage and tRNA abundance in higher eukaryotes is more complex. It is believed that codon usage and cellular tRNA pool coevolved for an accurate and efficient protein production (Bulmer, 1987; Shields, 1990; Duret, 2000). In support, highly expressed genes, e.g., those encoding ribosomal proteins, generally contain preferred codons which are recognized by abundant tRNA species. However, finding direct correlations between codon usage and tRNA abundance in multicellular eukaryotes is complicated due to heterologous structure of the genome (i.e., isochoric structure), variations in individual gene structure (the length of the coding sequence and introns), and higher tRNA gene redundancy (Hurst and Williams, 2000; Kanaya et al., 2001; Comeron, 2004; dos Reis et al., 2004; Semon et al., 2006).

Development of high-throughput tools, including tRNA-based microarrays (Dittmar et al., 2004; Polte et al., 2019), and more recently, optimized methods for high-throughput tRNA sequencing (Zheng et al., 2015; Gogakos et al., 2017; Shigematsu et al., 2017; Erber et al., 2020; Pinkard et al., 2020) have provided more efficient means for detection of individual tRNA-level fluctuations and enabled the discovery of key trends that regulate cell homeostasis in a tRNA-dependent way. These studies revealed that tissue-specific tRNA expression generally reflects codon preference of highly expressed genes in human, indicating the importance of translation regulation via tRNA abundance in different tissues (Dittmar et al., 2006). This is consistent with the idea that codon-mediated translation control can regulate tissue-specific protein expression (Plotkin et al., 2004). Some of the more complex trends have also been revealed, suggesting that abundance and expression of specific tRNAs can drive reprogramming of different cell states, such as proliferation, differentiation, and metastatic potential (Gingold et al., 2014; Goodarzi et al., 2016; Zhang et al., 2018b). Opposing tRNA-level signatures have been reported in proliferating and differentiating cells, and it has been found that changes in individual tRNA abundance correlate with, and potentially coordinate, selective gene expression profiles operating different states of the cell (Gingold et al., 2014). Elevated levels of individual tRNAs promote cells’ metastatic progression by enhancing stability and translation of metastasis-driven genes enriched for their cognate codons, thereby shifting the cellular program toward disease-promoting state (Goodarzi et al., 2016). Thus, changes in translation efficiencies driven by the combination of codon usage and tRNA abundance constitute a powerful mechanism of protein regulation.

For the last 50 years, tRNA has been studied exclusively as an adaptor molecule between mRNA nucleotide sequence and amino acids in protein synthesis. New studies provide evidence that tRNAs perform a number of previously unanticipated regulatory roles in various metabolic pathways, from modulation of global gene expression to regulation of cell death. In prokaryotes, tRNAs are known to be used in cell wall biosynthesis, antibiotic biogenesis, and transcription attenuation, i.e., via T-box mechanism [see (Katz et al., 2016) for a recent review of non-canonical roles of tRNAs in prokaryotes].

Here we discuss the roles tRNAs play in critical regulatory pathways, in addition to their conventional roles in translation, focusing largely on eukaryotic and mammalian systems.

## tRNAs Modulate Global Gene Expression

All cells are surrounded by a changing environment, which modulates cellular homeostasis and serves as a source of diverse forms of signals, including stress. Cells develop various mechanisms to respond to stress conditions, including reprogramming of gene expression that enables cells to adapt and maintain their normal physiology. Some of this reprogramming links directly to the protein synthesis machinery and tRNA.

Different types of stress trigger phosphorylation of serine 51 in the eukaryotic translation initiation factor 2α (eIF2α). This phosphorylation reduces the formation of the ternary complex (eIF2-GTP-Met-tRNA_i_^Met^) that transfers Met-tRNA_i_^Met^ to the ribosome, therefore leading to a decrease in global protein synthesis (Merrick and Pavitt, 2018). Four eIF2α kinases are regulated by different stimuli: protein kinase R-like ER kinase (PERK) by unfolded proteins in the ER, protein kinase R (PKR) by double-stranded RNA in virus-infected cells, heme-regulated inhibitor (HRI) by heme deficiency in erythroid cells, and general control non-repressible kinase 2 (GCN2) by serum starvation (Taniuchi et al., 2016; Wek, 2018).

**FIGURE 2:**
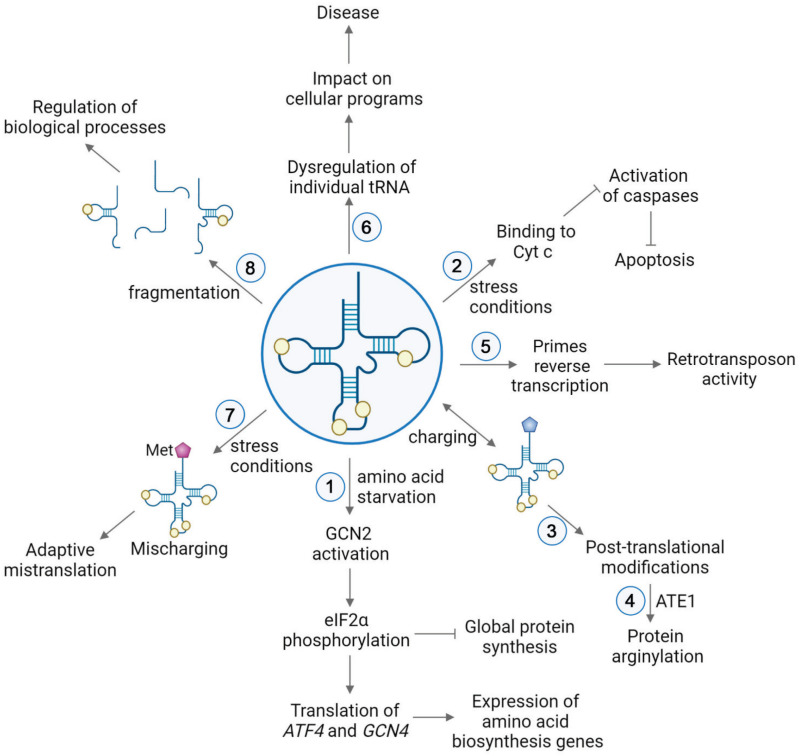
tRNA functions in various pathways in cell physiology. (1) Upon nutrient deficiency, binding of deacylated tRNAs to GCN2 leads to conformational changes of the kinase GCN2, resulting in phosphorylation of eIF2α. This causes repression of global protein synthesis, along with translation of activating transcription factor 4 (ATF4) in mammals and general control protein (GCN4) in yeast. These proteins induce selective expression of amino acid biosynthesis genes upon stress (Hinnebusch et al., 2016; Young and Wek, 2016). (2) Stress conditions trigger the release of cytochrome c (Cyt c) from mitochondria to the cytoplasm. In the cytoplasm, binding of tRNAs to Cyt c prevents association of Cyt c with Apaf-1. Thereby, the caspase cascade required for apoptosis is precluded (Mei et al., 2010; Suryanarayana et al., 2012; Gorla and Sepuri, 2014; Liu et al., 2016). (3) Aminoacyl-tRNAs serve as a donors in posttranslational modifications. (4) In protein arginylation, ATE1 transfers Arg from arginyl-tRNA^Arg^ onto a protein substrate. (5) tRNA is used as a primer in the reverse transcription of LTR retrotransposons (Martinez, 2017). (6) Dysregulation of individual tRNA contributes to the onset and severity of different diseases by impacting cellular programs. (7) Mismethylation of tRNAs is induced in response to stress conditions. Extra incorporation of Met in newly synthesized proteins serves an evolutionary strategy for expanding the genetic information in proteins (Netzer et al., 2009; Wiltrout et al., 2012; Lee et al., 2014; Wang and Pan, 2015; Schwartz et al., 2016). (8) Generation of tRNA-derived small RNAs leads to global regulatory responses (Keam and Hutvagner, 2015; Xie et al., 2020).

Cytoplasmic tRNAs rapidly translocate into the nucleus under a number of stress conditions, including nutrient starvation (Shaheen and Hopper, 2005; Takano et al., 2005; Hurto et al., 2007; Shaheen et al., 2007; Whitney et al., 2007; Dhakal et al., 2019), heat shock (Miyagawa et al., 2012; Watanabe et al., 2013), viral infection (Zaitseva et al., 2006), and oxidative stress (Schwenzer et al., 2019). This translocation not only directly depletes tRNAs from protein synthesis but also modulates stress response by activation of the GCN2 kinase (Castilho et al., 2014). GCN2 specifically binds deacylated tRNA via its histidyl-tRNA synthetase-like domain, which discriminates against aminoacyl-tRNAs (Dong et al., 2000; Zaborske et al., 2010; Figure 2). This binding leads to a conformational change of GCN2 and activates the kinase, which in turn phosphorylates eIF2α (Dever et al., 1992; Anda et al., 2017). Phosphorylated eIF2α is a competitive inhibitor of the guanine nucleotide exchange factor eIF2B (Adomavicius et al., 2019), and thus phosphorylation inhibits the recycling of eIF2 between the cycles of translation, resulting in downregulation of the global protein synthesis. Simultaneously, this pathway triggers increased translation of a select number of mRNAs containing short upstream open reading frames (uORF). Two of those mRNAs encode activating transcription factor 4 (ATF4) in mammals and general control protein (GCN4) in yeast (Hinnebusch et al., 2016; Young and Wek, 2016), which are required for selective expression of amino acid biosynthesis genes upon stress. Under normal conditions, translation of uORFs leads to repression of main ORFs (mORF) of *ATF4* and *GCN4*. During nutrient deficiency, phosphorylated eIF2α reduces the ternary complex concentration, which leads the 40S ribosomal subunit to bypass the uORF start codon, instead initiating translation at *ATF4* and *GCN4* mORFs (Somers et al., 2013; Hinnebusch et al., 2016).

Similar to mammalian cells, nutrient deprivation decreases amino acid availability and causes deacylated tRNA accumulation in prokaryotes (Potrykus and Cashel, 2008; Steinchen and Bange, 2016). The binding of deacylated tRNAs to the A-site of the ribosome induces the production of alarmones, referred to as (p)ppGpp, through the ribosome-associated protein RelA (Brown et al., 2016). In *E. coli*, (p)ppGpp directly binds to RNA polymerase and changes its affinity for sigma factors, leading to modulation of global gene expression (Ross et al., 2013, 2016). The binding of (p)ppGpp to RNA polymerase limits transcription of rRNA genes required for rapid growth while promoting the expression of stress response and amino acid biosynthesis genes. (p)ppGpp also binds to various proteins, leading to inhibition of cellular processes, such as translation and nucleotide biosynthesis (Kanjee et al., 2012; Wang et al., 2019). This in turn reprograms bacterial metabolism to slow down growth and reallocate resources during nutrient deficiency.

In addition to this generalized stress response, individual tRNAs also play regulatory roles in gene expression in humans. Misfolded tRNA^Asp^GUC isodecoder, referred to as tRNA^Asp^7, binds to Alu RNA element present in the 3′ UTR of the AspRS mRNA sequence (Rudinger-Thirion et al., 2011). This interaction alters 3′ UTR folding, thereby reshaping the accessibility of the two alternative polyadenylation sites and leading to induction of expression of AspRS (Rudinger-Thirion et al., 2011). Thus, a single tRNA isodecoder regulates translation of an essential enzyme in the cell.

## tRNAs Control Cell Death

Apoptosis is a cellular process of elimination of damaged or unwanted cells, characterized by distinct morphological changes, including membrane blebbing and nuclear fragmentation. Apoptosis plays major roles in normal physiology and disease and is considered a vital component of organismal homeostasis [see, e.g., (Tang et al., 2019) for a recent review]. This pathway can be initiated through one of the two routes: an extrinsic pathway, in which extracellular ligands bind to cell-surface death receptors, or an intrinsic pathway, which originates within the cell and is mediated by mitochondria (Pistritto et al., 2016). Both types of apoptosis are primarily executed by caspases (cysteinyl, aspartate-specific proteases) that induce global proteolysis and thus trigger cell death. There are two types of caspases: initiator caspases, e.g., caspase-8 and -9, which are activated by auto-processing, and effector caspases, e.g., caspase-3, -6, and -7, which become active upon proteolytic cleavage by initiator caspases (McIlwain et al., 2013).

**FIGURE 3:**
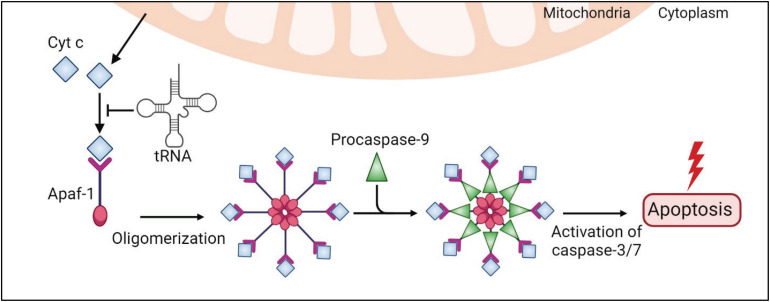
tRNAs play a protective role in the intrinsic apoptosis pathway. An internal stimulus, e.g., DNA damage or viral infection, causes the loss of mitochondrial membrane potential and leads to a release of cytochrome c (Cyt c) to the cytoplasm. Cytosolic Cyt c binds to the death adaptor apoptotic protease-activating factor-1 (Apaf-1) and ATP, triggering assembly of the oligomeric apoptosome complex, which recruits procaspase-9, leading to its activation. Caspase-9 activates effector caspases, e.g., caspase-3/7, and eventually leads to cell death. In this pathway, tRNAs directly bind to Cyt c and prevent Cyt c association with Apaf-1 and apoptosis.

The intrinsic apoptotic pathway is mediated by intracellular stimuli that converge at the mitochondrial level under various stress conditions, e.g., irradiation, viral infection, and oncogene activation. This pathway causes the loss of mitochondrial membrane potential, which leads to the release of cytochrome c (Cyt c) into the cytoplasm (Tait and Green, 2010; Figure 3). Cyt c is an essential mitochondrial protein which builds an electrochemical gradient driving ATP synthesis. In the cytoplasm, Cyt c binds to the death adaptor apoptotic protease-activating factor-1 (Apaf-1) and ATP or dATP, which triggers assembly of the oligomeric apoptosome complex (Acehan et al., 2002; Figure 3). This complex recruits procaspase-9, leading to its auto-proteolytic activation (Rodriguez and Lazebnik, 1999; Zou et al., 1999; Li et al., 2017; Figure 3). Then, caspase-9 activates effector caspases, e.g., caspase-3/7, and eventually degrades many cellular proteins and results in cell death (Figure 3).

Transfer RNAs have been shown to modulate apoptosis (Mei et al., 2010; Suryanarayana et al., 2012; Gorla and Sepuri, 2014; Liu et al., 2016; Figures 2, 3). Addition of RNase to cell extracts enhances Cyt c-induced caspase-9 activation, while exogenous cellular RNA supplementation reduces auto-activation of caspase-9 in a dose-dependent manner (Mei et al., 2010). Systematic analysis showed that tRNAs directly bind to Cyt c, leading to prevention of Cyt c association with Apaf-1 (Mei et al., 2010). Microinjection of tRNA into living cells inhibits apoptosis, while tRNA degradation enhances caspase activation (Mei et al., 2010). Taken together, these results show the critical role of tRNA in programmed cell death (Figures 2, 3).

Transfer RNAs bind to Cyt c through its heme domain, which defines the redox state of the protein (Suryanarayana et al., 2012; Gorla and Sepuri, 2014). It was proposed that tRNA interaction prevents the positively charged residues of Cyt c from being exposed to the Apaf-1 complex (Gorla and Sepuri, 2014). There are conflicting studies on how tRNA binding affects the redox state of Cyt c. Gorla and colleagues found that oxidized Cyt c is not able to interact with tRNA (Gorla and Sepuri, 2014). However, another study revealed that oxidized Cyt c can bind to tRNA with a 2-fold weaker affinity compared to the reduced form and, furthermore, that this binding promotes Cyt c reduction (Liu et al., 2016). Moreover, tRNA interaction hinders the peroxidase activity of Cyt c, which plays a role in the activation of the caspase cascade and Cyt c release from the mitochondria (Liu et al., 2016). Hence, this study concludes that tRNA is capable of regulating apoptosis by switching off the active form of Cyt c.

Recently, it has been found that Cyt c recognizes the L-shaped tertiary structure of tRNAs, particularly the core region (Liu et al., 2016). The binding affinity of Cyt c to tRNA is at a similar range to other tRNA-binding proteins [*K*_*d*_ 1–3.5 μM for Cyt c (Liu et al., 2016), compared to 1–3 μM for aaRS (Zhang et al., 2006; Liu et al., 2007, 2011) and 0.8–3.3 μM for tRNA nucleotidyltransferase (Kim et al., 2009)], therefore it is unknown how Cyt c competes with other tRNA-binding proteins *in vivo*.

Altogether, these results unveil an important role of tRNA in controlling cell death by preventing Cyt c from Apaf-1 activation. Notably, both tRNA and Cyt c are highly conserved molecules. Cyt c is a crucial mitochondrial protein that carries electrons from Cyt c reductase to Cyt c oxidase as part of the electron transport chain. This function has been evolutionarily conserved in eukaryotes over 1.5 billion years (Ow et al., 2008). Likewise, tRNA exists in all forms of life and its structure is identical in all organisms. Therefore, tRNA:Cyt c interaction has been proposed to be ancient (Hou and Yang, 2013; Raina and Ibba, 2014) and might have been established early in evolution as an indicator of the balance between protein synthesis, energy production, and cell survival.

## tRNAs Are Utilized for Protein Modifications

Posttranslational protein modifications are fundamental processes that significantly amplify the repertoire of the proteome. Covalent addition of various chemical groups to proteins plays essential roles in physiological and pathological processes. In addition to a number of groups that are different from any elements of protein structure, proteins can be extensively modified by posttranslational addition of amino acids (Kaji et al., 1963; Kaji, 1968; Fung and Fahlman, 2015; Gadadhar et al., 2017; He et al., 2018; Kimura et al., 2018; Chen and Kashina, 2019). Some of these modifications involve direct amino acid transfer; however, a specific class of these modifications, including arginylation in eukaryotes (Kaji et al., 1963; Kaji, 1968; Chen and Kashina, 2019), L/F transfer in prokaryotes (Fung and Fahlman, 2015), and the recently demonstrated amino acid addition to the primary amines of the Lys side chains (He et al., 2018), involve transfer of amino acids from the charged tRNA. Here we overview arginylation, a posttranslational modification of emerging importance that regulates fundamental mechanisms in eukaryotes and mammalian cells (Figure 2).

Arginylation was discovered more than 50 years ago, when researchers observed addition of radioactively labeled amino acids to proteins in tRNA-dependent but ribosome-independent manner (Kaji et al., 1963; Kaji, 1968). Arginylation is mediated by arginyltransferase 1 (ATE1) that transfers arginine from aminoacyl-tRNAs onto proteins. Initial studies revealed that ATE1 targets N-terminally exposed acidic residues (Glu and Asp) of proteins (Soffer, 1971). Cys was also found to be arginylated in mammalian cells (Gonda et al., 1989). More recently, it has been shown that Cys oxidization greatly facilitates its arginylation, and this mechanism has been proposed to mediate intracellular oxygen sensing in mammalian cells (Hu et al., 2005) and plants (Gibbs et al., 2011). Lately, it was shown that arginylation is not limited to N-terminal residues; mid-chain Glu and Asp residues were found to be arginylated on their side chains in intact proteins (Wang et al., 2014).

Arginylation is essential for regulation of physiological functions of key proteins *in vivo*. Ate1 deletion in mice leads to embryonic lethality and severe defects in cardiovascular development and angiogenesis (Kwon et al., 2002). N-terminal arginylation of β-actin facilitates cell motility and was proposed as a mechanism that contributes to cell migration *in vivo* (Karakozova et al., 2006; Kurosaka et al., 2010). Arginylated calreticulin regulates stress granules scaffolding and enhances apoptotic response (Carpio et al., 2013; Comba et al., 2019). Moreover, lack of arginylation leads to neurodegeneration (Wang et al., 2017). Altogether, arginylation acts as a global regulator to control various biological conditions in cells (Saha and Kashina, 2011; Rassier and Kashina, 2019).

Although arginylation was discovered almost 60 years ago, its molecular mechanism is not fully elucidated. Our recent study shows that ATE1-mediated arginylation is highly specific to tRNA^Arg^ conjugated with Arg (Avcilar-Kucukgoze et al., 2020). Mouse tRNA^Arg^ species can all participate in arginylation, albeit with somewhat different efficiencies, suggesting potential *in vivo* preferences of ATE1 enzyme for specific tRNA^Arg^ (Avcilar-Kucukgoze et al., 2020). However, it is difficult to estimate whether these preferences amount to different efficiencies of arginylation by different tRNAs *in vivo*, since tRNA repertoire is highly dynamic (Torrent et al., 2018). On the other hand, abundance of arginyl-tRNA^Arg^ also depends on availability of intracellular arginine, which, in addition to its role in protein synthesis, also serves as a precursor for the synthesis of important molecules such as nitric oxide, urea, polyamines, proline, glutamate, creatine, and agmatine (Szefel et al., 2019). Thus, it is possible that intracellular level of Arg plays a key role in balancing all these processes. In support, numerous cancers are defective in arginine biosynthesis due to reduced expression of arginine biosynthesis enzymes, argininosuccinate synthetase, and argininosuccinate lyase (Karatsai et al., 2020). Arg deprivation has been directly linked to impairments in actin arginylation and cell migration in invasive glioblastomas (Pavlyk et al., 2015). Ate1 deletion results in carcinogenic transformation of cultured fibroblasts (Rai et al., 2016). Hence, deficiency of intracellular Arg may affect fundamental processes, potentially due to altered arginyl-tRNA^Arg^ availability.

## tRNA Prime Reverse Transcription of Retroviruses and Retrotransposons

Retrotransposons are mobile genetic elements abundantly found in eukaryotic genomes. Their transposition mechanism involves an RNA intermediate. Based on this mechanism, retrotransposons are divided into two groups: (1) Long terminal repeat (LTR) retrotransposons and (2) non-LTR retrotransposons (Mita and Boeke, 2016).

Long terminal repeat retrotransposons are characterized by the repeats of a few hundred base pairs on both ends. They replicate via reverse transcription using a cytoplasmic tRNA, with a sequence complementary to the primer binding site (PBS) at the 5′ end, acting as a primer. After reverse transcription, RNA template is partially degraded and full-length dsDNA is integrated into the chromosomal DNA [see (Martinez, 2017) for the recent review of tRNA role in retrotransposition]. LTR retrotransposons are structurally and evolutionary related to retroviruses, and the mechanism of LTR transposon replication via tRNA-primed reverse transcription and genome integration resembles that used by the retroviruses. The difference between retroviruses and retrotransposons is the PBS size. While PBS size is 18 nts in retroviruses, it varies between 8 and 18 nts in retrotransposons (Wilhelm and Wilhelm, 2001).

Specific tRNAs utilized as primers for different retrotransposons and retroviruses are determined by their PBS. For example, human immunodeficiency virus type 1 (HIV-1) uses human tRNA^Lys,3^ (Wain-Hobson et al., 1985), HTLV-1 uses tRNA^Pro^ (Seiki et al., 1983) while retrotransposons of the copia group use mostly tRNA^Met^ as a primer (Kikuchi et al., 1986). In the case of HIV-1, human tRNA^Lys,3^ is selectively packaged into the virus particles and the level of tRNA^Lys,3^ in the virion correlates with the level of viral infectivity (Gabor et al., 2002). Lysyl-tRNA synthetase (LysRS) has been also found in HIV-1 virions, and it has been suggested that its interaction with the viral structural proteins Gag and Gag-Pol is required for tRNA^Lys,3^ encapsidation. It has been suggested that phosphorylation of LysRS on Ser207 promotes its release from the multisynthetase complex and its association with Gag (Duchon et al., 2017). Another study found that only mitochondrial LysRS (mLysRS) is present in HIV-1 extracts (Kaminska et al., 2007). Recently, the molecular details of the interaction between mLysRS and Gag-Pol have been revealed (Phongsavanh et al., 2020). The C-terminal integrase subunit of Gag-Pol is shown to stabilize the tRNA^Lys,3^:mLysRS interaction (Khoder-Agha et al., 2018; Phongsavanh et al., 2020).

## tRNAs Play Emerging Roles in Disease

Above and beyond its role in viral infection, recent studies have proved that the fluctuation of tRNA repertoire plays a crucial role in shaping the cellular proteomic landscape. Dysregulation of individual tRNAs contributes to the onset and severity of various diseases, including metastatic and non-metastatic cancers, Huntington disease, cystic fibrosis, seizures, multiple myeloma, and neurodegeneration (Pavon-Eternod et al., 2009, 2013; Zhou et al., 2009; Girstmair et al., 2013; Ishimura et al., 2014; Birch et al., 2016; Goodarzi et al., 2016; Kirchner et al., 2017; Zhang et al., 2018b; Kapur et al., 2020; Figure 2).

The activity of PolIII is known to be upregulated in cancer, providing sufficient tRNA abundance to support protein synthesis in rapidly proliferating cells (Kantidakis et al., 2010; Arimbasseri and Maraia, 2016; Sriskanthadevan-Pirahas et al., 2018). tRNA levels are elevated up to 10 fold in breast cancer and multiple myeloma cell lines (Pavon-Eternod et al., 2009; Zhou et al., 2009). Interestingly, the increase of tRNAs is not uniform. tRNA^Arg^CCU, tRNA^Arg^UCU, tRNA^*Thr*^CGU, tRNA^Leu^UAA, tRNA^*Tyr*^GUA, and tRNA^*Ser*^GCU are more strongly overexpressed than others (Pavon-Eternod et al., 2009). tRNA^*Glu*^UUC and tRNA^Arg^CCG have been found to promote breast cancer metastasis (Goodarzi et al., 2016). The upregulation of these tRNAs increases translation efficiency of disease-promoting genes, which are enriched in the corresponding codons (Goodarzi et al., 2016). tRNA^Arg^ was reported to be pervasively upregulated in eight cancer types, and tRNA^Asn^ was upregulated in five cancer types (Zhang et al., 2018b). Moreover, elevated level of tRNA_i_^Met^ was reported to boost metabolic activity and proliferation in various cancer cells by reshaping the entire tRNA landscape (Pavon-Eternod et al., 2013; Gingold et al., 2014; Birch et al., 2016). Interestingly, the tRNA_i_^Met^ level is regulated posttranscriptionally by miR-34a, which suppresses breast carcinogenesis and inhibits the proliferation of breast cancer cells (Wang et al., 2018). Thus, tRNA_i_^Met^ has been proposed to be an oncogene by itself (Wang et al., 2018). Collectively, these studies suggest that individual tRNA expression is involved in tumor progression [see (Santos et al., 2019) for additional examples of tRNA deregulation in cancer].

Accumulating evidence supports the importance of tRNA expression in shaping various cellular programs. tRNA repertoire influences the onset and severity of diseases in a tissue-specific manner by modulating translation of specific genes. Kirchner and coworkers studied the impact of a specific synonymous single-nucleotide polymorphism (SNP) in the cystic fibrosis transmembrane conductance regulator (CFTR), which substitutes Thr ACT codon to ACG codon (Kirchner et al., 2017). This synonymous SNP alters protein stability and function. Researchers revealed that ACG codon is encoded by low-abundance tRNA^*Thr*^CGU, particularly in human bronchial epithelia, which leads to slower local translation speed at Thr854 codon (Kirchner et al., 2017). The elevated level of tRNA^*Thr*^CGU rescues the effects of synonymous SNP and restores the CFTR protein conformation and function (Kirchner et al., 2017). tRNA^*Gln*^CUG is implicated in Huntington’s disease (HD) (Girstmair et al., 2013), caused by extensive repeat of Gln CAG codon in the protein of huntingtin. Expression of an expanded CAG stretch depletes tRNA^*Gln*^CUG, leading to translational frameshifting and marked changes in the aggregation of huntingtin protein *in vivo* (Girstmair et al., 2013). Thus, depletion of a single tRNA may change translation dynamics and aggravate a major disease phenotype.

Mutations in tRNA genes cause many diseases. Two examples of best-characterized syndromes linked to mt-tRNA mutations are myoclonic epilepsy and ragged-red fiber (MERRF), and mitochondrial encephalomyopathy, lactic acidosis, and stroke-like episodes (MELAS) (Kirino and Suzuki, 2005). Majority of MELAS and MERRF cases are caused by single-point mutations [A3243G or T3271C for MELAS (Goto et al., 1990, 1991), A8344G for MERRF (Shoffner et al., 1990)] of mt-tRNA^Leu^UAA and mt-tRNA^Lys^UUU, respectively. These pathogenic mutations hinder the taurine modification (5-taurinomethyluridine, i.e., τm^5^U, for mt-tRNA^Leu^UAA and 5-taurinomethyl-2-thiouridine, i.e., τm^5^s^2^U, for mt-tRNA^Lys^UUU) in the wobble position (U34). While wild-type mt-tRNA^Leu^UAA decode both UUA and UUG codons, the wobble modification deficiency in MELAS disrupts the decoding of UUG codon due to destabilization of U:G wobble base pairing (Yasukawa et al., 2000). This decoding bias specifically reduces the translation of the UUG-enriched NADH dehydrogenase 6 gene (*MT-ND6*), which is the component of the mitochondrial complex I. Similarly, in the case of MERRF, a 50–60% decrease in mt-tRNA^Lys^UUU aminoacylation capacity results in severe protein synthesis impairment due to premature termination of translation at Lys codons (AAA and AAG) (Enriquez et al., 1995). The pathological mutations are found at different locations within the tRNA molecule outside the anticodon, and it appears that they disturb the recognition of the RNA-modifying enzymes, which are responsible for producing the 5-taurinomethyl and 2-thio groups of the wobble bases of the mt-tRNAs (Koga et al., 2012). Since uridine modifications at the wobble position are crucial for precise and efficient codon recognition (Novoa et al., 2012; Agris et al., 2018), mutant mt-tRNAs induce translational defects of cognate codons and cause considerable decoding disorders. Considering that tRNA functions require multiple posttranscriptional modifications (Pan, 2018), it is expected that tRNA modification disorders develop widely in human diseases. Chronic ophthalmoplegia, cardiomyopathy, and hypertension are some examples of diseases caused by mutant mt-tRNAs. A complete list in MITOMAP (Brandon et al., 2005) and an extensive list of such diseases are included in Boczonadi and Horvath (2014), Kirchner and Ignatova (2015), Tahmasebi et al. (2018), Schaffer et al. (2019).

In mice, a point mutation (C50T in T-stem loop) of the central nervous system (CNS)-specific nuclear-coded tRNA^Arg^UCU isodecoder (*n-Tr20*) results in a significant reduction of aminoacylation level and ribosome stalling at the AGA codons, although mouse genome contains four other tRNA^Arg^UCU genes (Ishimura et al., 2014). tRNA^Arg^ C50T variants, including in the UCU isoacceptor, have also been observed in humans (Lant et al., 2019); however, no evidence has been found that these variants cause human disease. It is elusive how the expression of a single tRNA isodecoder is regulated in a tissue-specific manner, because the promoter sequences used by PolIII are identical among the five members of the tRNA^Arg^UCU family. Interestingly, loss of wild-type (wt) *n-Tr20* alters signaling pathways regulating transcription and translation, leading to changes in synaptic transmission and reduced seizure susceptibility (Kapur et al., 2020). Deletion of wt *n-Tr20* regulates the transcription of 236 genes and splicing of 377 genes, demonstrating the role of a single tRNA isodecoder at different stages of RNA metabolism (Kapur et al., 2020).

## Regulated tRNA-Dependent Mistranslation Serves as Adaptation Mechanism Upon Stress

For many decades since the original discovery of translation, it has generally been assumed that translational machinery is optimized to maintain translation errors at a minimum to sustain the cellular fitness. However, regulated mistranslation through tRNA misacylation may serve a beneficial mechanism to adapt the stress conditions (Figure 2). While technically translation-dependent, this role of tRNAs is unconventional enough to merit inclusion into this review.

Stress conditions induce tRNA misacylation with Met in mammalian cells (Netzer et al., 2009; Lee et al., 2014; Wang and Pan, 2015), yeast (Wiltrout et al., 2012), and bacteria (Jones et al., 2011; Schwartz et al., 2016; Schwartz and Pan, 2017). In mammalian cells, mismethionylated tRNAs constitute ∼1% compared to methyl-tRNAs^Met^ under normal conditions; however, Met-misacylation increases up to 10 fold upon oxidative stress (Netzer et al., 2009). Mammalian tRNA mismethionylation is regulated through phosphorylation of two Ser residues of MetRS by the extracellular signal-regulated kinase (ERK) (Lee et al., 2014). The incorporation of Met at non-Met codons has been detected in various proteins (Netzer et al., 2009; Wiltrout et al., 2012; Lee et al., 2014; Wang and Pan, 2015), indicating that mismethionylated tRNAs are directly utilized in translation. Extra-genetic incorporation of Met residues protects enzyme active sites against reactive oxygen species (ROS)-mediated damage through their reactive sulfur group (Levine et al., 1996; Luo and Levine, 2009). Upon Ca^+2^ stress, extra incorporation of Met has been found in Ca^+2^/calmodulin-dependent protein kinase II (CaMKII), a multifunctional protein required for cellular Ca^+2^ homeostasis (Wang and Pan, 2015). Met-mistranslated CaMKII shows elevated activity especially when the cells are treated with Ca^+2^ (Wang and Pan, 2015). Met-mutant CaMKII proteins have distinct expression levels and subcellular localizations compared to the wild type protein under Ca^+2^ stress (Wang and Pan, 2015). Moreover, human AlaRS mischarges tRNA^Cys^ through recognition of G4:U69 base pair in the acceptor stem, resulting in Cys-to-Ala substitution in the overexpressed reporter protein in HEK293T cells (Sun et al., 2016). The biological meaning of this AlaRS mischarging mechanism is unknown (Sun et al., 2016), but studies suggest that such regulated tRNA misacylation represents an evolutionary strategy for expanding the genetic information in proteins, especially under stress conditions.

## Generation of tRNA Fragments Serves Regulatory Roles *in vivo*

Earlier studies reported the existence of stable tRNA-derived small RNAs (tsRNA), presumably generated by cleavage of mature tRNA (Borek et al., 1977; Speer et al., 1979). Initially, these tsRNAs have been assumed to be by-products of random degradation of tRNAs, but recent advances in high-throughput sequencing methods revealed the existence of numerous stable tsRNAs in all three domains of life. A growing body of evidence unveils diverse biological roles of tsRNAs in cellular physiology and suggests that these molecules constitute a class of small non-coding RNAs involved in global cellular regulation (Keam and Hutvagner, 2015; Xie et al., 2020; Figure 2).

**FIGURE 4:**
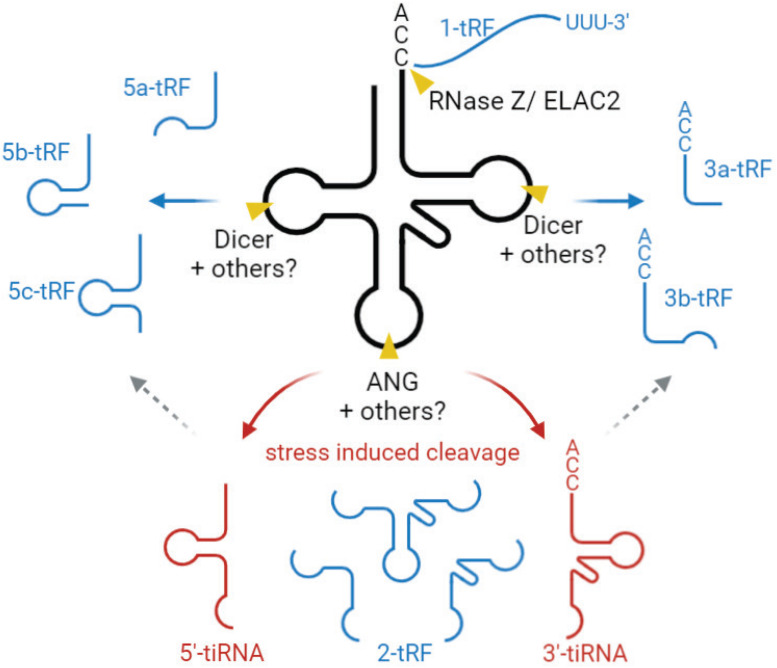
Biogenesis and classification tsRNAs. tsRNAs are divided into two main types, tiRNA (red) and tRF (blue). tiRNAs are produced by a specific cleavage at the anticodon loop of mature tRNA, generating two fragments, 5′-tiRNA and 3′-tiRNA. This cleavage is mediated by angiogenin (ANG), mostly under stress conditions. 5- and 3-tRF are produced by a specific cleavage at or near D- and T-loops of mature tRNAs. “a,” “b,” and “c” for 5-tRFs and 3-tRFs to define fragments of different length (∼15–18, 22, and 31 nt, respectively). The enzymes mediating this cleavage are not fully characterized but are believed to involve endonuclease Dicer and/or RNase T2. 2-tRF is derived from the internal region and generated by the unknown cleavage method. 1-tRF contains 3′-trailer, produced by RNase Z/ELAC2. tsRNA, tRNA-derived small RNA, tiRNA, stress induced tRNA fragment, tRF, tRNA-derived fragment.

Based on their length and the tRNA cleavage sites involved in their generation, tsRNAs are mainly divided into two groups. One is stress-induced tRNA fragments (tiRNAs), produced by a specific cleavage at or near the anticodon loop, generating two fragments of 31–40 nts in length (Xie et al., 2020; Figure 4). In mammals, this cleavage is mediated by angiogenin (ANG) [a member of the ribonuclease (RNase) A superfamily], generating 5′- and 3′-tRNA halves (Fu et al., 2009; Yamasaki et al., 2009; Elkordy et al., 2018; Su et al., 2019; Figure 4). In yeast, tRNA halves are generated by Rny1p (a member of RNase T2 family) (Thompson and Parker, 2009). tiRNA accumulation is generally induced by various stress conditions, e.g., nutrition deficiency, UV radiation, heat shock, and oxidative stress (Thompson et al., 2008; Fu et al., 2009; Yamasaki et al., 2009; Elkordy et al., 2018).

The second group is tRNA-derived fragments (tRFs), produced by a specific cleavage at or near D- and T-loops, yielding fragments of 14–30 nt in length, corresponding to 5′-derived tRFs (5-tRF) and 3′-derived tRFs (3-tRF) (Figure 4), as well as the internal piece corresponding to the tRNA sequence in between (2-tRF, also referred to as tRF-i) (Telonis et al., 2015) and a 3′-trailer (1-tRF). 5-tRF and 3-tRF come at different lengths, and thereby these fragments are further classified into 5a-tRFs (∼15 nt), 5b-tRFs (∼22 nts), 5c-tRFs (∼31 nts), 3a-tRFs (∼18 nt), and 3b tRFs (∼22 nt) (Kumar et al., 2015). tRNA cleavage generating these fragments is believed to be mediated by endonuclease Dicer, a member of the RNase III family (Babiarz et al., 2008; Cole et al., 2009; Yeung et al., 2009; Haussecker et al., 2010; Maute et al., 2013); however, other studies show that Dicer deficiency does not abolish tRF generation, suggesting that other RNase(s) are also involved (Li et al., 2012; Kumar et al., 2014; Kuscu et al., 2018). In plants, it has been proposed that RNase T2, rather than Dicer, is the main enzyme generating these regulatory tRFs (Megel et al., 2019), and it is possible that RNAse T2 also acts in this pathway in other organisms. Overall, the questions of potential additional enzymes involved in this process, and their hierarchy *in vivo*, require further investigation. It appears likely that different RNases can mediate tRF generation under different conditions to modulate tRF-regulated processes in the cell.

Transfer RNA modifications have an impact on the tsRNAs biogenesis. NSun2 and Dnmt2 mediate posttranscriptional methylation of tRNA at cytosine-5 (m^5^C) (Bohnsack et al., 2019). Loss of mammalian Dnmt2 and NSun2 resulted in hypomethylated tRNAs and accumulation of tsRNAs (Tuorto et al., 2012; Blanco et al., 2016; Zhang et al., 2018a). It was found that lack of m^5^C at C38 position significantly changes the RNA secondary structure (Zhang et al., 2018a), which might facilitate tRNA cleavage. Highly expressed PUS7 catalyzes the modification of uridine (U) to pseudouridine and its deletion in human embryonic stem cells affects specific tRFs, especially those derived from tRNA containing a 5′ terminal oligoguanine (TOG) (Guzzi et al., 2018). These results show that RNA modifications contribute to the generation of tsRNA. tsRNAs levels can vary in different cells and tissues, as well as in different diseases; therefore, several databases listing the entire repertoire of cellular tsRNAs have been generated (Kumar et al., 2015; Selitsky and Sethupathy, 2015; Zheng et al., 2016; Pliatsika et al., 2018; Li et al., 2020; Yao et al., 2020; Zuo et al., 2020). Potentially, modulation of tsRNA levels can be involved in disease-related cellular reprogramming and constitute conceptually novel targets for therapeutics.

**FIGURE 5:**
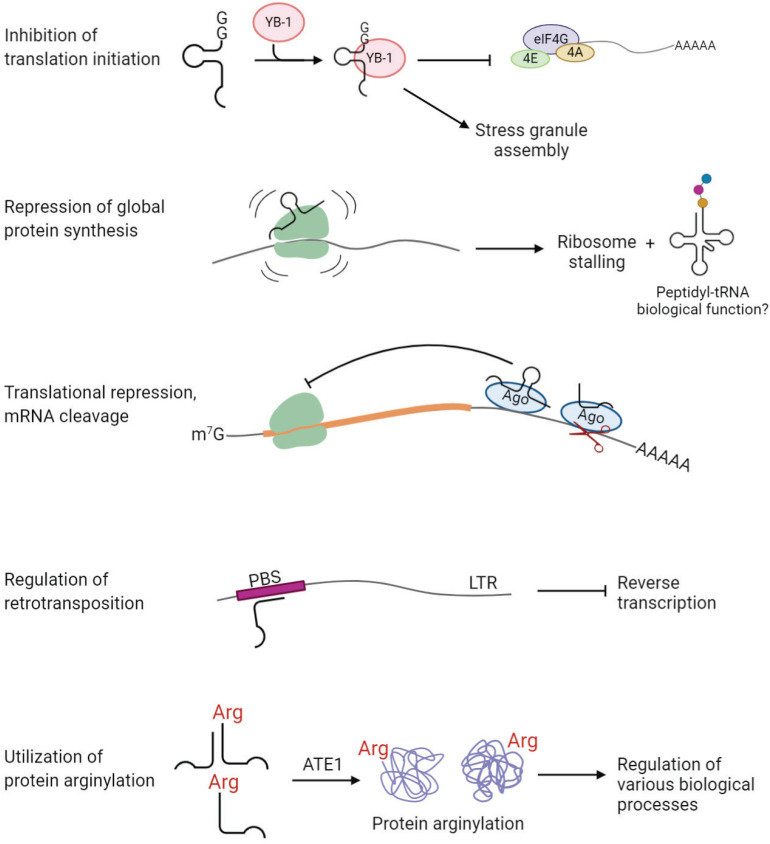
tsRNAs play regulatory functions in cell physiology. Examples of tsRNA functions include inhibition of translation initiation, repression of global protein synthesis, translational repression and mRNA cleavage, regulation of retrotransposition, and utilization of protein arginylation.

Stress-induced tRNA halves, especially 5′-tiRNAs, are known to reduce global protein synthesis by ∼20% (Yamasaki et al., 2009). This reduction cannot be a direct consequence of depletion of mature cytoplasmic tRNA pool, because only a small fraction (<5%) of total tRNA is fragmented upon stress (Fu et al., 2009; Yamasaki et al., 2009; Saikia et al., 2012). Evidence suggests that tiRNAs, upon cleavage, cause translational repression. It was found that 5′-tiRNAs, but not 3′-tiRNAs, derived from tRNA^Ala^ and tRNA^Cys^, inhibit translation initiation (Ivanov et al., 2011). 5′-tiRNAs interact with the cold shock domain of the translation repressor Y-box-binding protein 1 (YB-1), which leads to displacement of cap-binding complex eIF4F from the cap structures (m^7^G) (Ivanov et al., 2011; Figure 5). tiRNA-induced translation inhibition also induces the assembly of stress granules, which constitute translationally stalled mRNAs, associated pre-initiation factors, and signaling proteins (Emara et al., 2010; Ivanov et al., 2011). 5′-tiRNA^Ala/Cys^ possess a unique oligoguanine motif at their 5′ terminus, which forms G-quadruplex (G4) structures required for translation inhibition (Ivanov et al., 2014). On the other hand, tsRNAs can directly interact with the ribosomes and inhibit global protein synthesis in various organisms (Gebetsberger et al., 2012; Sobala and Hutvagner, 2013; Gonskikh et al., 2020). A recent study suggests that 5′-tiRNA^Pro^, produced in a stress-independent manner, represses global protein synthesis through binding to the ribosomes in mammalian cells (Gonskikh et al., 2020; Figure 5). Interestingly, addition of 5′-tiRNA^Pro^ leads to accumulation of peptidyl-tRNA inside the arrested ribosomes (Gonskikh et al., 2020; Figure 5). It is unknown whether the peptidyl-tRNA product possesses a biological role by itself or if it is a by-product of stalled ribosomes (Gonskikh et al., 2020). Moreover, some tsRNAs have functions similar to known miRNAs and piRNAs, repressing expression of a target gene by binding to complementary sites in 3′ UTR.

Several studies revealed an association of tsRNAs with Argonaute (Ago) proteins, the key players in RNA silencing (Cole et al., 2009; Haussecker et al., 2010; Li et al., 2012; Kumar et al., 2014; Kuscu et al., 2018; Guan et al., 2020) [reviewed in Shigematsu and Kirino (2015)] (Figure 5). In HeLa cells, Dicer-dependent 5′-tRF^*Gln*^CUG is associated with Ago1 and Ago2, even though the binding is weak (Cole et al., 2009). Another study showed that 1-tRFs and 3-tRFs preferentially bind to Ago3/4 over Ago1/2 in HEK293 cells (Haussecker et al., 2010). 3-tRF^His^GTG and 3-tRF^Leu^CAG were detected in Ago2-IP fractions in MEFs (Li et al., 2012). Analysis of PAR-CLIP data revealed that both 5-tRFs and 3-tRFs bind to Ago1, 3, and 4, but not Ago2, in HEK293 (Kumar et al., 2014). Moreover, 3-tRFs derived from tRNA^Leu^AAG, tRNA^Cys^GCA and tRNA^Leu^TAA downregulate expression of luciferase reporters in Ago-dependent manner (Kuscu et al., 2018). Importantly, the elevated level of 3-tRF^Leu^TAA significantly decreases the expression of target genes *in vivo* in HEK293T cells (Kuscu et al., 2018). A recent study found that 3-tRF^Ala^AGC interacts with Ago2 to modulate the expression of tumor suppressor FBZO47 by binding to 3′ UTR, resulting in enhanced cell proliferation, migration and invasion in gastric cancer (Zhang et al., 2020). Altogether, accumulating evidence suggests that tsRNAs are capable of repressing target genes posttranscriptionally through binding to Ago proteins, similar to miRNAs.

Some, recent studies classify the roles of tsRNAs in translational regulation into two global pathways: Ago-dependent, which targets specific mRNAs to induce translation inhibition, and Ago-independent, which acts by exerting structural effects on mRNA and rRNA independently of the Ago proteins. These aspects of tsRNA-dependent translation regulation have been recently reviewed in Shi et al. (2019).

The protective role of tRNAs against apoptosis, described earlier in this review, also involves tRNA-derived fragments. A recent study showed that the released Cyt c preferentially binds ANG-induced tiRNAs, but not full-length tRNAs, in response to hyperosmotic stress in mouse embryonic fibroblasts, leading to attenuation of Apaf-1 oligomerization and apoptosome activity (Saikia et al., 2014). This report points that tiRNAs also play a critical role in the activation of the apoptosis pathway under stress.

An interesting study published in 2017 has unveiled a “safeguard” role of tsRNAs in genome integrity in mammalian cells (Schorn et al., 2017; Figure 5). LTR retrotransposons integrated into the genome are tightly regulated to prevent mutations, especially during early embryonic development, when epigenetic suppression of transposon elements is inconceivable. This study found that abundant endogenous 3-tRFs can specifically bind tRNA primer binding site (PBS) at the 5′ UTR of LTR retrotransposons, where tRNA normally binds during LTR retrotransposon replication (Schorn et al., 2017). These 3-tRFs repress retrotranspositions of the two most active mouse transposons by inhibiting reverse transcription in preimplantation stem cells (Schorn et al., 2017). It is still unknown how the cell arranges silencing of transposable elements by generating tRFs in certain conditions.

The role of tsRNA in intercellular communication is only beginning to be explored. tsRNAs are enriched in extracellular vehicles (EVs) more than any other class of RNA (Chiou et al., 2018). For T cells, 45% of tRFs are at least 1.5-fold enriched in EVs; some are enriched up to 10-fold (Chiou et al., 2018). Interestingly, silencing of EV-enriched tRFs promotes T cell activation, suggesting that these tRFs are actively released by secretion to prevent repression of immune activation (Chiou et al., 2018). tRNA halves can be delivered to the sperm during its maturation in epididymis, potentially via EVs (Sharma et al., 2016, 2018). These tRNA halves regulate embryonic gene expression of metabolic pathways depending on parental diet (Chen et al., 2016; Sharma et al., 2016). Stable 5′ tRNA halves are found to circulate in mouse blood, most likely as part of a nucleoprotein complex (Dhahbi et al., 2013). A recent study unraveled the generation of tRNA halves in the extracellular environment by RNase 1, a highly active secreted nuclease, after the release of full-length tRNAs into the extracellular space (Nechooshtan et al., 2020). However, their cellular targets are not yet known (see (Tosar and Cayota, 2020) for a recent review of extracellular tRNA-derived fragments).

In principle, amino acid-charged tRNAs can be cleaved by tsRNA-generating machinery to produce aminoacyl-tsRNAs. Such aminoacyl-tsRNAs would then be incapable of participating in translation, due to the lack of anticodon loop, but can potentially still serve as amino acid donors for other reactions utilizing tRNA-bound amino acids. Given that tRNA secondary and tertiary structure is generally very stable, it appears possible that after tRNA cleavage, 5-tRF and 3-tRFs stick together due to the complementary base pairing. While this has never been directly demonstrated, some examples of amino acid-charged tsRNAs have been identified. A previous study found abundant aminoacylated tiRNAs derived from tRNA^Asp^GUC, referred to as SHOT-RNAs, in breast and prostate cancers (Honda et al., 2015). Our recent study showed that arginyl-tRF^Arg^ can be generated *in vitro* from Arg-tRNA^Arg^ using RNase T2 and that such Arg-tRF^Arg^ can mediate arginylation (Avcilar-Kucukgoze et al., 2020; Figure 5). As translation and arginylation compete for the same substrate, arginyl-tRNA^Arg^, such aminoacyl-tRF generation in this case, if it happens *in vivo*, may serve as a switch between these two processes (Avcilar-Kucukgoze et al., 2020). In support, lack of Ate1 significantly alters the ratio of tRNA^Arg^:tRF^Arg^ in MEFs, suggesting a functional link between tRF^Arg^ and arginylation *in vivo* (Avcilar-Kucukgoze et al., 2020). We propose that arginyl-tRF^Arg^ generation is a mechanism that maintains the balance between translation and arginylation and can potentially shift this balance in favor of one or the other process in response to physiological stimuli. This study illustrates that aminoacyl-tRFs may serve as amino acid donors in pathways other than translation, and opens up a possibility of other pathways that depend on the regulated balance between translation and ribosome-independent utilization of tRNA-conjugated amino acids.

## Conclusion

Emerging data from multiple studies suggest that, in addition to the tRNAs’ role in translation, these small non-coding RNAs play a wealth of other functions that regulate and fine-tune multiple processes in normal physiology and disease. These studies so far have just scratched the surface of the complexity involved in the biological functions of tRNAs. In this review, we described the usage of tRNAs in various mechanisms and pathways, which are critical for the fate of the cell. Some of these processes involve uncharged tRNA as a regulator; however, some of them consume aminoacyl-tRNA which is also a substrate for translational machinery. It is an important question how ribosome and other aminoacyl-tRNA-dependent processes compete for the common substrate, i.e., aminoacyl-tRNAs. For example, L/F transferase has a strong preference for the most abundant tRNA^Leu^CAG (Fung et al., 2014). However, under starvation, the tRNA^Leu^CAG aminoacylation level drops to 5% (Dittmar et al., 2005). How does L/F transferase compete with the ribosome, especially when the leucyl-tRNA^Leu^ gets scarce? In fact, it is a general question for all of the processes which rely on the use of charged tRNAs. Although it remains largely elusive, tRNA fragmentation may serve a mechanism that potentially shifts the balance depending on the demand of the cell for either translation or arginylation (Avcilar-Kucukgoze et al., 2020). On the other hand, tRNAs with specific posttranscriptional modifications may be dedicated to particular non-translational processes. There is no doubt that development of new tools will provide insights into the competition for the aminoacyl-tRNAs in the future.

## Author Contributions

Both authors conceptualized, planned, and wrote the manuscript.

## Conflict of Interest

The authors declare that the research was conducted in the absence of any commercial or financial relationships that could be construed as a potential conflict of interest.
